# Supplementary material for: 233U/236U signature allows to distinguish environmental emissions of civil nuclear industry from weapons fallout
Source: Nat Commun. 2020 Mar 9;11:1275. doi: 10.1038/s41467-020-15008-2 (PMC7062840; doi:10.1038/s41467-020-15008-2)
Supplement: Supplementary file 2 — Supplementary Information [file 41467_2020_15008_MOESM2_ESM.pdf]

Supplementary information

$^{233}U/^{236}U$ signature allows to distinguish environmental emissions of civil nuclear industry from weapons fallout

Hain et al.

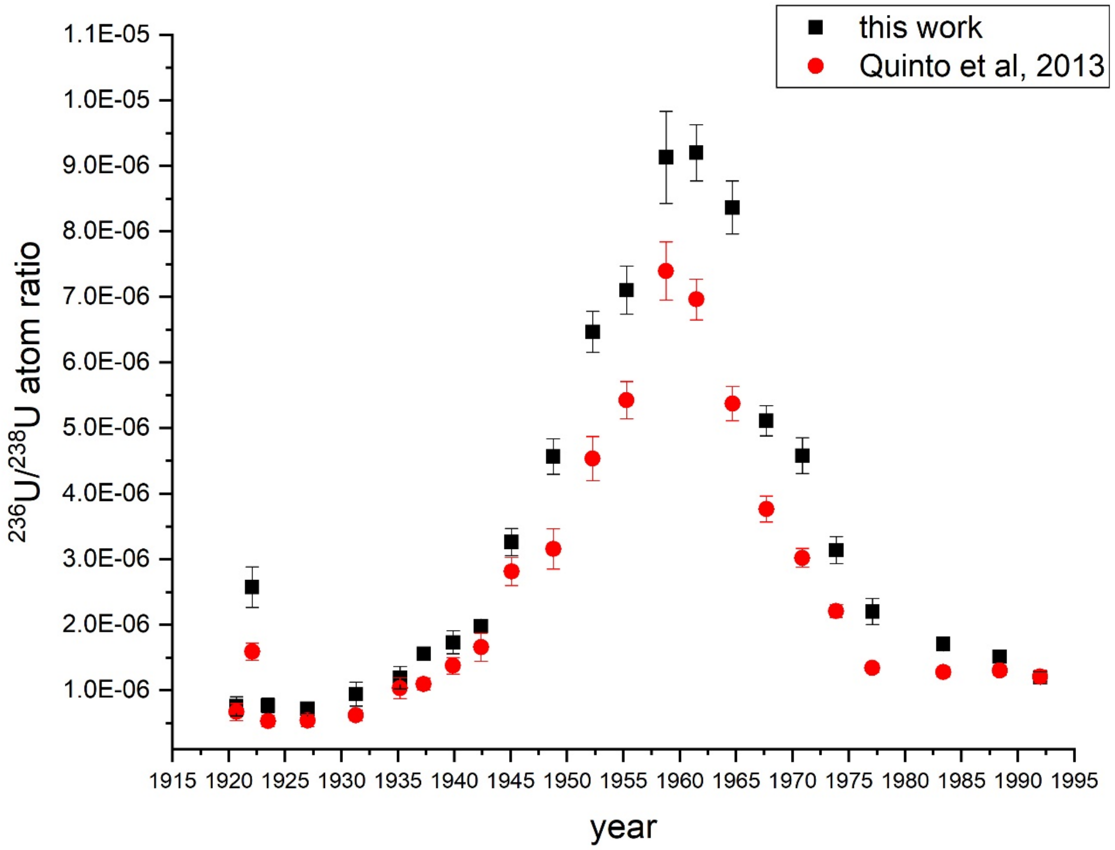

Supplementary Figure 1: Illustration of the measurement results for the $^{236}U/^{238}U$ atom ratio obtained in the present study (black squares) and published in Quinto et al., 2013 [1] (red dots) for comparison. Uncertainties are given in ±1 σ (s.e.m). The deviation between the two measurements must have been caused by a different detection efficiency for the two isotopes ^{236}U and ^{234}U in the previous measurement. With the present procedure such a deviation would be detected by the standard material and corrected for. The conclusions drawn in [1] are not affected by the absolute value of the $^{236}U/^{238}U$ ratio.

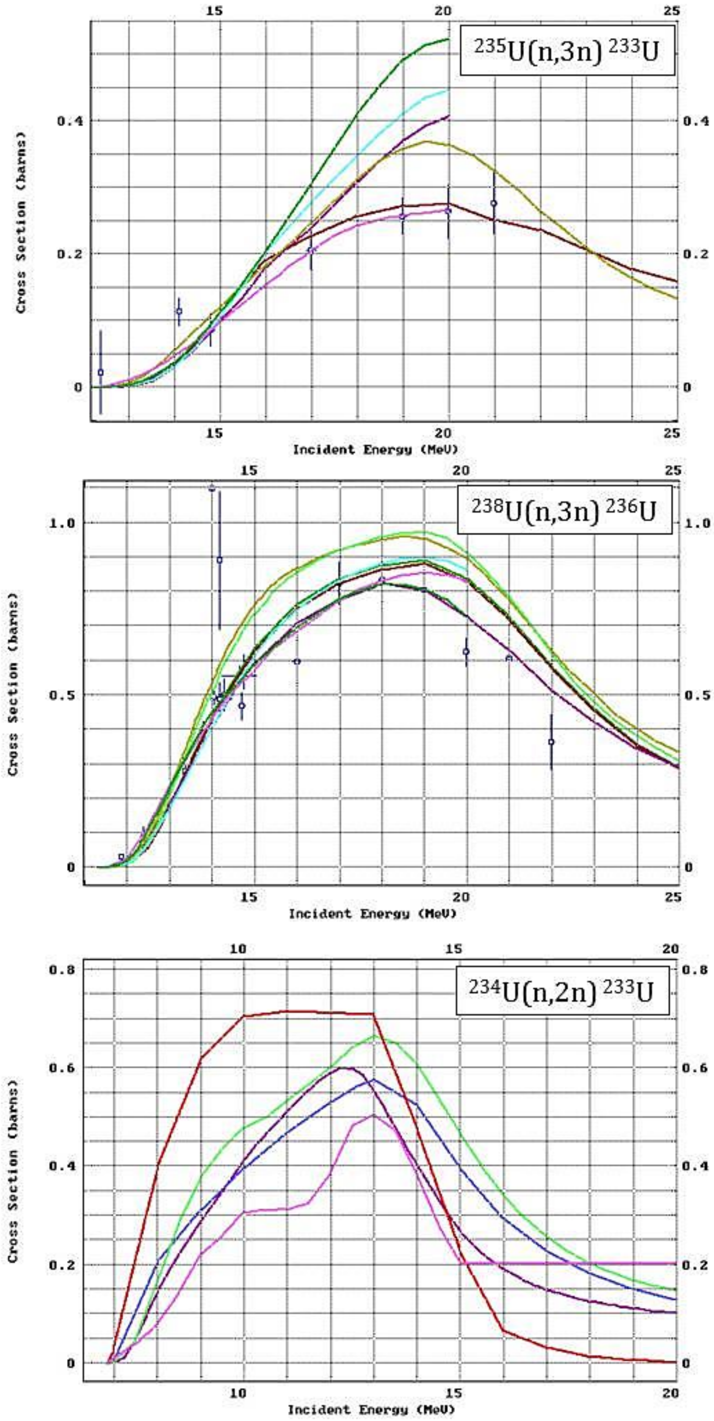

Supplementary Figure 2. Cross-sections for the ^{235}U(n,3n)^{233}U, the ^{238}U(n,3n)^{236}U, and ^{234}U(n,2n)^{233}U reactions from different nuclear model calculations (solid lines) including experimental data (blue squares). Obtained from the ENDF/EXFOR database [2, 3].

Age of the peat layer (AD)	^{238}U (ppm) ± 5% [1]	^{233}U counts	$^{233}U/^{238}U$ (10^{-9})	$^{236}U/^{238}U$ (10^{-8}) this work	$^{236}U/^{238}U$ (10^{-8}) [1]	$^{233}U/^{236}U$ (10^{-2})
1920.7 ± 1.2	0.042	12	11 ± 3	75 ± 15	67 ± 13	1.5 ± 0.6
1922.1 ± 1.2	0.05	26	17 ± 3	257 ± 31	159 ± 13	0.7 ± 0.2
1923.5 ± 1.2	0.063	22	13 ± 3	77 ± 11	53 ± 9	1.7 ± 0.4
1927.0 ± 1.1	0.057	43	38 ± 7	72 ± 8	53 ± 10	5.5 ± 1.2
1931.3 ± 1.0	0.088	19	17 ± 4	94 ± 18	62 ± 8	1.8 ± 0.4
1935.2 ± 0.9	n.a.	69	45 ± 6	119 ± 17	103 ± 16	3.7 ± 0.6
1937.3 ± 0.9	0.086	103	47 ± 6	157 ± 8	109 ± 9	2.9 ± 0.5
1939.9 ± 0.9	0.105	140	60 ± 9	173 ± 17	137 ± 13	3.5 ± 0.7
1942.4 ± 0.8	0.103	186	71 ± 5	198 ± 10	166 ± 21	3.6 ± 0.4
1945.1 ± 0.8	0.084	182	96 ± 10	326 ± 21	281 ± 21	2.9 ± 0.6
1948.8 ± 0.8	n.a.	276	144 ± 16	457 ± 27	316 ± 31	3.2 ± 0.4
1952.3 ± 0.7	0.094	507	173 ± 12	647 ± 31	453 ± 33	2.7 ± 0.2
1955.3 ± 0.7	0.148	625	178 ± 7	711 ± 37	543 ± 28	2.5 ± 0.2
1958.8 ± 0.7	0.102	222	106 ± 7	913 ± 71	740 ± 44	1.2 ± 0.1
1961.5 ± 0.6	n.a.	452	100 ± 8	920 ± 43	696 ± 31	1.1 ± 0.1
1964.7 ± 0.6	0.069	147	58 ± 6	837 ± 41	537 ± 26	0.7 ± 0.1
1967.7 ± 0.6	0.053	98	27 ± 4	511 ± 23	377 ± 20	0.5 ± 0.1
1970.9 ± 0.6	n.a.	105	30 ± 4	458 ± 27	302 ± 14	0.6 ± 0.1
1973.9 ± 0.5	0.048	120	17 ± 2	314 ± 20	221 ± 10	0.5 ± 0.1
1977.1 ± 0.5	0.042	28	11 ± 2	220 ± 20	134 ± 7	0.5 ± 0.1
1983.4 ± 0.5	0.04	23	11 ± 2	170 ± 9	128 ± 8	0.6 ± 0.1
1988.4 ± 0.5	0.042	22	12 ± 3	151 ± 7	130 ± 8	0.8 ± 0.2
1992.0 ± 0.5	n.a.	25	13 ± 3	119 ± 9	121 ± 10	1.1 ± 0.2

Supplementary Table 1. Individual measurement results of $^{236}U/^{238}U$, $^{233}U/^{238}U$ and $^{233}U/^{236}U$ for the ombrotrophic peat core. Results for $^{236}U/^{238}U$ are compared to the previously published data [1]. Uncertainties are given in ±1 σ (s.e.m). Unsupported ^{210}Pb dating was used to determine the age of the layers. For samples with high $^{236}U/^{238}U$ ratios in some cases the results from the present and the previous study do not agree within 2 σ uncertainty. $^{236}U/^{238}U$ ratios from the current measurement are generally higher than the values published in [1]. This indicates a systematic deviation which is not included in the uncertainties listed in the table for ref. [1] which only comprise statistical fluctuations. The observed discrepancies might be due to an improved measurement and data evaluation procedure used in the present study regarding the normalization of the results to the external standard samples. Systematic variations are better taken into account by the new procedure. A background contribution due to UH^{3+} can be excluded by monitoring the count rate on mass 239 from the in-house ^{236}U standard Vienna-KkU. An upper limit of the $239/^{238}U$ atom ratio of $8.1\cdot10^{-11}$ was measured. By scaling with the ^{238}U current of the samples (maximum 1.2 pA) relative to the Vienna-KkU and the natural abundance of ^{235}U, a maximum contribution of $^{235}UH^{3+}$ to the ^{235}U count rate of $3\cdot10^{-7}$ is obtained. This value is 6 orders of magnitude lower than the smallest count rate from a peat bog sample which demonstrates

that the results of the present work are not affected by the mentioned background. For the measurements presented in [1], charge state 5+ was chosen. In this case, molecular compounds are completely destroyed by the stripping process in the tandem accelerator.

Country	Year	Test Site	Test series	Name	Total yield [Mt]
USA	1952	Enewetak	Ivy	Mike	10.4
	1954	Bikini	Castle	Bravo	15
	1954	Bikini	Castle	Romeo	11
	1954	Bikini	Castle	Union	6.9
	1954	Bikini	Castle	Yankee	13.5
	1956	Bikini	Redwing	Navajo	4.5
	1956	Bikini	Redwing	Tewa	5.0
	1958	Enewetak	Hardtack I	Oak	8.9
	1958	Bikini	Hardtack I	Poplar	9.3
	1962	Christmas Island	Dominic	Bighorn	7.65
	1962	Johnston Island	Dominic	Housatonic	8.3
USSR	1961	Novaya Zemlya	-	Test 113	4
	1961	Novaya Zemlya	-	Test 114	4
	1961	Novaya Zemlya	-	Test 123	12.5
	1961	Novaya Zemlya	-	Test 130	50
	1961	Novaya Zemlya	-	Test 131	5
	1962	Novaya Zemlya	-	Test 147	21.1
	1962	Novaya Zemlya	-	Test 158	4
	1962	Novaya Zemlya	-	Test 160	4.2
	1962	Novaya Zemlya	-	Test 168	4
	1962	Novaya Zemlya	-	Test 173	19.1
	1962	Novaya Zemlya	-	Test 174	24.2
	1962	Novaya Zemlya	-	Test 219	24.2
China	1976	Lop Nor	-	-	4

Supplementary Table 2: List of atmospheric thermonuclear weapons tests with total explosion yields higher than 4 Mt (TNT equivalent) in chronological order. Taken from [4, 5].

Year [AD]	^{238}U (ppm) [6]	^{233}U counts	$^{233}U/^{238}U$ (10^{-11})	$^{236}U/^{238}U$ (10^{-9}) this work	$^{236}U/^{238}U$ (10^{-9}) [6]	$^{233}U/^{236}U$ (10^{-2})
1940	3.26 ± 0.06	1	< 0.2	0.13 ± 0.02	0.12 ± 0.02	< 2.7
1941	3.05 ± 0.03	0	< 0.2	0.10 ± 0.02	0.09 ± 0.01	< 1.9
1942	3.42 ± 0.06	0	< 0.4	0.13 ± 0.04	0.14 ± 0.03	< 3.4
1943	3.24 ± 0.04	2	1.4 ± 1.2	0.08 ± 0.05	0.09 ± 0.01	< 34
1944	3.18 ± 0.01	0	< 0.9	0.05 ± 0.05	0.06 ± 0.01	< 17
1945	2.97 ± 0.03	0	< 0.2	0.11 ± 0.03	0.08 ± 0.01	< 2.0
1946	3.26 ± 0.08	0	< 0.2	0.08 ± 0.03	0.08 ± 0.01	< 3.0
1947	3.51 ± 0.02	2	0.3 ± 0.2	0.10 ± 0.02	0.12 ± 0.03	2.8 ± 2.5
1948	3.10 ± 0.10	2	0.6 ± 0.5	0.09 ± 0.03	0.15 ± 0.02	6.7 ± 6.2
1949	3.27 ± 0.03	1	< 0.5	0.08 ± 0.02	0.14 ± 0.02	< 6.3
1950	3.24 ± 0.04	2	0.5 ± 0.4	0.22 ± 0.04	0.16 ± 0.03	< 4.3
1951	3.10 ± 0.02	1	< 0.5	0.35 ± 0.05	0.30 ± 0.07	< 1.4
1952	2.99 ± 0.04	4	0.6 ± 0.3	1.40 ± 0.08	1.30 ± 0.10	0.4 ± 0.2
1953	3.07 ± 0.06	8	1.8 ± 0.7	3.23 ± 0.18	3.64 ± 0.16	0.5 ± 0.2
1954	3.10 ± 0.03	17	2.4 ± 0.6	10.5 ± 0.5	11.0 ± 1.2	0.2 ± 0.1
1955	3.53 ± 0.12	6	14.1 ± 6.2	6.02 ± 0.68	8.55 ± 0.44	2.3 ± 1.1
1956	3.21 ± 0.04	17	2.8 ± 0.7	4.57 ± 0.19	4.60 ± 0.32	0.6 ± 0.2
1957	3.14 ± 0.05	42	7.4 ± 1.2	4.13 ± 0.18	4.44 ± 0.26	1.8 ± 0.3
1958	3.25 ± 0.07	49	15.7 ± 2.3	7.80 ± 0.27	8.55 ± 1.17	2.0 ± 0.3
1959	3.20 ± 0.02	13	8.8 ± 2.5	6.59 ± 0.38	7.72 ± 0.45	1.3 ± 0.4
1960	3.28 ± 0.04	29	8.0 ± 1.7	4.62 ± 0.22	4.17 ± 0.31	1.7 ± 0.4
1961	3.41 ± 0.11	19	5.5 ± 1.3	3.11 ± 0.16	3.48 ± 0.35	1.8 ±0.4
1962	3.12 ± 0.14	19	5.6 ± 1.3	2.74 ± 0.18	2.58 ± 0.33	2.0 ± 0.5
1963	3.25 ± 0.02	18	4.9 ± 1.2	3.08 ± 0.16	3.51 ± 0.20	1.6 ± 0.4
1964	3.33 ± 0.05	29	4.7 ± 0.9	3.20 ± 0.13	3.18 ± 0.30	1.5 ± 0.3
1965	3.22 ± 0.04	22	4.2 ± 0.9	2.83 ± 0.27	3.23 ± 0.16	1.5 ± 0.4
1966	3.33 ± 0.06	30	4.0 ± 0.7	2.82 ± 0.15	2.88 ± 0.14	1.4 ± 0.3
1967	3.32 ± 0.07	11	4.1 ± 1.3	2.71 ± 0.17	2.73 ± 0.17	1.5 ± 0.5
1968	3.34 ± 0.03	19	3.5 ± 0.8	2.58 ± 0.12	2.46 ± 0.13	1.4 ± 0.3
1969	3.28 ± 0.10	25	3.5 ± 0.7	2.54 ± 0.19	2.41 ± 0.14	1.4 ± 0.3
1970	3.22 ± 0.08	14	3.1 ± 0.9	2.19 ± 0.22	2.35 ± 0.13	1.4 ± 0.4

Supplementary Table 3: Individual measurement results of $^{236}U/^{238}U$, $^{233}U/^{238}U$ and $^{233}U/^{236}U$ for the Kume coral core. Results for $^{236}U/^{238}U$ are compared to the previously published data. Uncertainties are given in ± 1 σ (s.e.m.). The data published in [6] is already blank corrected which leads to small deviations for samples with small $^{236}U/^{238}U$ ratios, i.e. samples before 1950. For samples with an uncertainty larger than the measurement value, only an upper limit for the $^{233}U/^{238}U$ and thus, also for $^{233}U/^{236}U$ ratio, can be derived.

Depth (cm)	Diluted 1:100	^{238}U (ppb)	^{233}U counts	$^{233}U/^{238}U$ (10^{-9})	$^{236}U/^{238}U$ (10^{-6}) this work	$^{236}U/^{238}U$ (10^{-6}) [7]	$^{233}U/^{236}U$ (10^{-2})
3*	no		1664	9.6 ± 0.2	8.77 ± 0.06	8.15 ± 0.66	0.109 ± 0.003
11*	yes		17	19 ± 5	5.67 ± 0.50	9.22 ± 0.71	0.33 ± 0.10
19	no		18424	128 ± 2	51.0 ± 0.6	43.6 ± 2.6	0.251 ± 0.005
23	yes		39	32 ± 6	25.9 ± 1.2	38.2 ± 2.3	0.12 ± 0.02
35	no		5679	46.3 ± 0.6	39.3 ± 0.6	32.9 ± 4.0	0.118 ± 0.002
39	no		4842	58.9 ± 0.9	43.1 ± 2.6	34.5 ± 3.7	0.136 ± 0.008
47	yes		32	50 ± 1	27.7 ± 1.7	29.6 ± 4.1	0.18 ± 0.04
IAEA-381	-	2.9 ± 0.3 [8]	87	2.2 ± 0.3	1.99 ± 0.03	2.04 ± 0.02 [9]	0.11 ± 0.01

Supplementary Table 4: $^{236}U/^{238}U$, $^{233}U/^{238}U$ and $^{233}U/^{236}U$ results of the Irish Sea sediment sample and the reference material IAEA-381 (Irish Sea water). Uncertainties are given in ±1σ (s.e.m.). Results for $^{236}U/^{238}U$ are compared to the previously published data [7]. *A different aliquot from the same material than in ref. [7] was used which can lead to an increased discrepancy between the $^{236}U/^{238}U$ ratios obtained in the present study and the previously published results. In addition, during the previous measurements, the ion source was operated at an exceptionally low temperature to arrive at an acceptable ^{236}U count rate in the detector. This was not required for the present study and might be a reason for additional deviations between the $^{236}U/^{238}U$ results from the two studies.

Supplementary References:

[1] F. Quinto, E. Hrnecek, M. Krachler, W. Shotyk, P. Steier, and S. R. Winkler. Measurements of ^{236}U in Ancient and Modern Peat Samples and Implications for Postdepositional Migration of Fallout Radionuclides. *Environ. Sci. Technol.* **47,** 5243-5250, (2013).

[2] Chadwick, M. B. et al. ENDF/B-VII.1: Nuclear data for science and technology: cross sections, covariances, fission product yields and decay data. Nucl. Data Sheets (2011). Database Version of 2017-03-06, www-nds.iaea.org/exfor/endf.htm.

[3] Otuka, N. et al. Towards a more complete and accurate experimental nuclear reaction data library (EXFOR): International collaboration between Nuclear Reaction Data Centres (NRDC). Nucl. Data Sheets (2014). Database Version of 2017-03-02, www-nds.iaea.org/exfor/exfor.htm.

[4] United Nations Scientific Committee on the Effects of Atomic Radiation (UNSCEAR). *Sources and Effects of Ionizing Radiation, UNSCEAR 2000 Report, Volume I: Sources (ANNEX C)*. (United Nations Publication, New York, USA, 2000).

[5] S. L. Simon and W. L. Robinson. A Compilation of Nuclear Weapons Test Detonation Data for U.S. Pacific Ocean Tests. *Health Phys*. **73**, 258-264 (1997).

[6] T. Nomura et al. Reconstruction of the temporal distribution of $^{236}U/^{238}U$ in the Northwest Pacific Ocean using a coral core sample from the Kuroshio Current area. *Mar. Chem*., **190**, 28-34 (2017).

[7] M. Srncik, E. Hrnecek, P. Steier, and G. Wallner. Determination of U, Pu and Am isotopes in Irish Sea sediment by a combination of AMS and radiometric methods. *J. Environ. Radioact.* **102**, 331-335 (2011).

[8] M. K. Pham et al. A certified reference material for radionuclides in the water sample from Irish Sea (IAEA-443), *J. Radioanal. Nucl. Chem.* **288**, 603–611 (2011).

[9] R. Eigl, M. Srncik, P. Steier, and G. Wallner. $^{236}U/^{238}U$ and $^{240}Pu/^{239}Pu$ isotopic ratios in small (2 L) sea and river water samples. *J. Environ. Radioact.* **116**, 54-58 (2013).
